# Microwave-Assisted Solvothermal Synthesis of Cesium Tungsten Bronze Nanoparticles

**DOI:** 10.3390/nano15080627

**Published:** 2025-04-20

**Authors:** Jingyi Huang, Na Ta, Fengze Cao, Shuai He, Jianli He, Luomeng Chao

**Affiliations:** College of Science, Inner Mongolia University of Science and Technology, Baotou 014010, China; huang1999@stu.imust.edu.cn (J.H.); 2022023352@stu.imust.edu.cn (N.T.); 2019984@imust.edu.cn (F.C.); heshuaii@sina.com (S.H.)

**Keywords:** cesium tungsten bronze, nanocrystals, synthesis, NIR shielding

## Abstract

Cesium tungsten bronzes (Cs_x_WO_3_), as functional materials with excellent near-infrared shielding properties, demonstrate significant potential for applications in smart windows. However, traditional synthesis methods, such as solid-state reactions and solvothermal/hydrothermal approaches, typically require harsh conditions, including high temperatures (above 200 °C), high pressure, inert atmospheres, or prolonged reaction times. In this study, we propose an optimized microwave-assisted solvothermal synthesis strategy that significantly reduces the severity of reaction conditions through precise parameter control. When benzyl alcohol was employed as the solvent, Cs_x_WO_3_ nanoparticles could be rapidly synthesized within a relatively short duration of 15 min at 180 °C, or alternatively obtained through 2 h at a low temperature of 140 °C. However, when anhydrous ethanol, which is cost-effective and environmentally friendly, was substituted for benzyl alcohol, successful synthesis was also achieved at 140 °C in 2 h. This method overcomes the limitations of traditional high-pressure reaction systems, achieving efficient crystallization under low-temperature and ambient-pressure conditions while eliminating safety hazards and significantly improving energy efficiency. The resulting materials retain excellent near-infrared shielding performance and visible-light transparency, providing an innovative solution for the safe, rapid, and controllable synthesis of functional nanomaterials.

## 1. Introduction

Tungsten oxide is an important semiconductor ceramic functional material with great potential for applications in fields such as electrochromism and photocatalytic degradation of pollutants [1,2]. When other cations are doped into tungsten oxide, its semiconductor properties usually transform into metallic conductivity, and other properties, such as optical characteristics, also undergo significant changes. For example, the insertion of cesium ions into the lattice of tungsten trioxide can form cesium tungsten bronzes (Cs_x_WO_3_). As a class of non-stoichiometric compounds with hexagonal tunnel structures, Cs_x_WO_3_ have garnered significant attention due to their unique electronic properties and near-infrared (NIR) absorption capabilities [3,4]. These materials exhibit exceptional optical characteristics, including high transparency in the visible region and strong shielding of NIR radiation, making them ideal candidates for applications such as smart windows, solar energy management, photo-thermal therapy, photocatalysis, gas sensors, and energy-efficient optical devices [5,6,7,8,9,10]. Their ability to selectively block heat-generating infrared rays while maintaining visible light transparency positions them as critical components in reducing energy consumption for climate control systems, thereby contributing to carbon emission mitigation [11].

Conventional synthesis methods for Cs_x_WO_3_, such as solid-state reactions [12,13,14,15], often require harsh conditions, including prolonged high-temperature treatments (≥600 °C) and inert or reductive atmospheres. These processes typically yield inhomogeneous particles with large grain sizes, which compromise their optical performance. Solvothermal or hydrothermal methods, as conventional techniques for preparing nanoparticles, have been widely used by many researchers to synthesize cesium tungsten bronze nanoparticles [16,17,18,19,20]. However, these methods typically require high-pressure reactors operating at temperatures above 200 °C, with reaction times often exceeding several hours. Although these methods can produce nanoparticles with small sizes and uniform distributions, the use of high-pressure reactors still poses certain safety risks. Researchers have attempted alternative methods, such as microwave-assisted solvothermal synthesis, but these reactions still demand elevated temperatures (200 °C) and extended durations (3–9 h) [21].

In this work, we demonstrate a green, low-temperature strategy that significantly advances the sustainability and efficiency of Cs_x_WO_3_ synthesis. By optimizing precursor dispersion and microwave irradiation parameters, our approach achieves phase-pure crystallization at 140 °C—representing a significant reduction in thermal energy input compared with previous methods—while simultaneously shortening the reaction time to 15 min at 180 °C. Our innovation addresses the critical need for safer, faster, and scalable production routes for functional nanomaterials, paving the way for broader industrial adoption of Cs_x_WO_3_-based technologies.

## 2. Materials and Methods

### 2.1. Reagents

Cesium hydroxide hydrate (analytical grade, 99.9%) and tungsten chloride (99.9%) were purchased from Macklin Biochemical Co., Ltd., Shanghai, Chian; benzyl alcohol (AR) was obtained from Zhiyuan Chemical Reagent Co., Ltd., Tianjin, China; ammonia solution (AR) was supplied by Yongfei Chemical Reagent Co., Ltd., Langfang, China; and anhydrous ethanol (AR) and sulfuric acid (AR) were acquired from Beilian Fine Chemical Development Co., Ltd., Tianjin, China; and Kelong Chemical Co., Ltd., Chengdu, China respectively. All chemicals were used as received without further purification.

### 2.2. Preparation of Cs_x_WO_3_ Using Benzyl Alcohol as Solvent

First, 0.399 g of hydrated cesium hydroxide and 2.855 g of tungsten chloride were dissolved in 50 mL of benzyl alcohol solution and stirred continuously for 40 min using a magnetic stirrer. The well-mixed precursor solution was then transferred into a 100 mL reaction vessel of a microwave synthesizer (XH-800A, Xianghu Technology Development Co., Ltd., Beijing, China, with a microwave frequency of 2450 MHz). The solution was heated at different temperatures of 120 °C, 140 °C, and 180 °C for 15 min, 30 min, 1 h, and 2 h, respectively. After natural cooling, the resulting blue precipitate was collected by centrifugation. The precipitate was washed sequentially with dilute sulfuric acid solution, deionized water, and anhydrous ethanol. Finally, the washed precipitate was dried under vacuum at 40 °C for 3 h, yielding the desired powder.

### 2.3. Preparation of Cs_x_WO_3_ Using Anhydrous Ethanol as Solvent

First, 0.3879 g of hydrated cesium hydroxide and 2.7759 g of tungsten chloride were dissolved in 50 mL of anhydrous ethanol solution and stirred continuously for 40 min using a magnetic stirrer. The pH of the precursor solution was adjusted to 1.5, 2.0, 2.5, 3.0, 3.5, 4.0, and 5.0, respectively. The solution was then heated at a synthesis temperature of 140 °C for 2 h using a microwave synthesizer. Then, following the same procedure, the precursor solutions for several other groups were prepared with the pH adjusted to 3.0 and heated at 140 °C and 120 °C for 15 min, 30 min, 1 h, and 2 h. After natural cooling, the precipitate was collected by centrifugation. The precipitate was washed three times with deionized water and anhydrous ethanol, respectively. Finally, the washed precipitate was dried under vacuum at 60 °C overnight, yielding the desired powder.

### 2.4. Fabrication Process for Cs_x_WO_3_ Coated Glass

Cs_x_WO_3_-coated glass was fabricated using the spin coating technique. Initially, 0.2 g of Cs_x_WO_3_ was sonicated in 20 mL of ethanol for 30 min to create a uniform dispersion. The sonication process was performed using an ultrasonic cleaner (Model: KS-100VDB/2, Jielimei Ultrasonic Instruments Co., Ltd., Kunshan, China) operating at a frequency of 45–80 kHz. Next, 5 g of polyvinyl butyral (PVB) resin was introduced into the mixture and stirred vigorously for 20 min to form the coating slurry. The slurry was then applied onto the surface of soda-lime glass substrates (10 × 10 cm, 1 mm thickness) through spin coating at a centrifugal speed of 2000 rpm for 40 s. Finally, the coated glass was placed in an oven at 40 °C for 1 h to ensure the removal of any remaining solvent.

### 2.5. Characterization

The phase identification of the sample was performed using X-ray diffraction (XRD, Smartlab SE, Rigaku, Tokyo, Japan) with a Cu Kα target (λ = 1.5406 Å), operating at 40 kV and 40 mA, with a scan rate of 1° per minute. The morphology of the sample was observed using a field emission scanning electron microscope (SEM, SU8010, Hitachi High-Tech, Tokyo, Japan) equipped with energy-dispersive spectroscopy (EDS, Ultim Max, Oxford Instruments, Abingdon, UK). The microstructure of the nanoparticles was further characterized using transmission electron microscopy (TEM, Tecnai G2 F30, FEI Company, Hillsboro, OR, USA) at an accelerating voltage of 300 kV. The oxidation states of the elements were analyzed using X-ray photoelectron spectroscopy (XPS, Escalab 250Xi, Thermo Fisher Scientific, Waltham, MA, USA) with a monochromatic Al Kα X-ray source. The transmittance of the coated glass was measured at room temperature using a UV-Vis-NIR spectrophotometer (UV-Vis, UH4150, Hitachi High-Tech, Tokyo, Japan).

## 3. Results and Discussion

### 3.1. Using Benzyl Alcohol as Solvent

**Figure 1:**
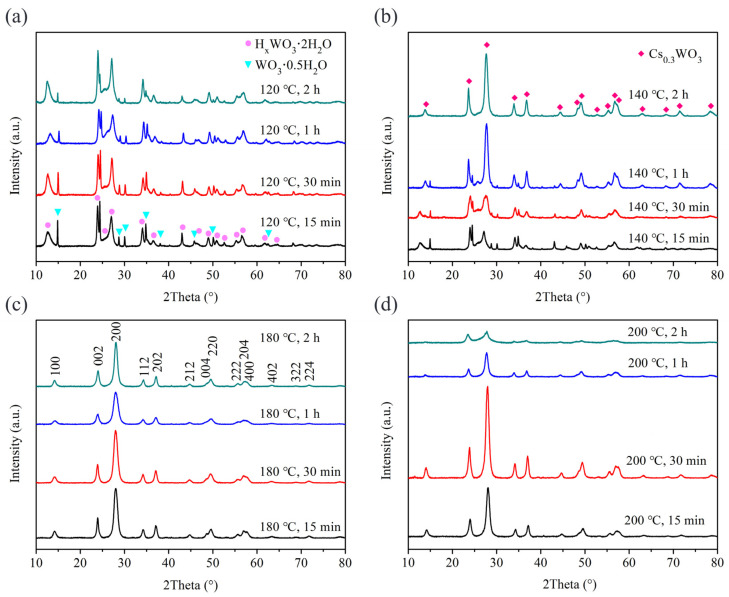
XRD patterns of samples using benzyl alcohol as the solvent: (**a**) different treatment time at 120 °C; (**b**) different treatment time at 140 °C; (**c**) different treatment time at 180 °C and (**d**) different treatment time at 200 °C.

**Figure 2:**
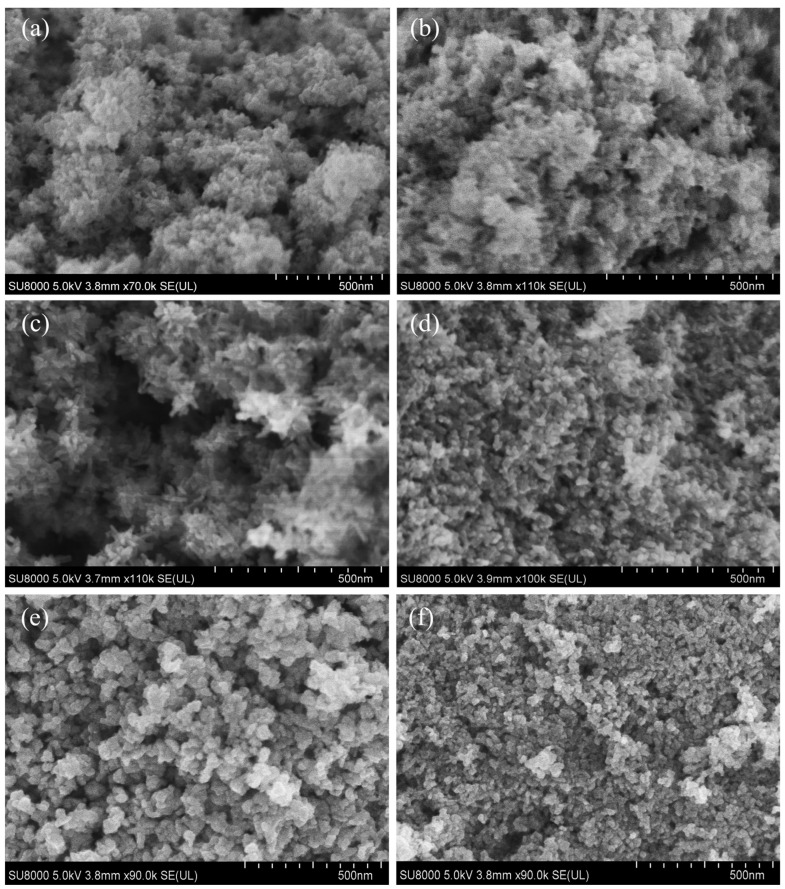
SEM images of six samples using benzyl alcohol as solvent: (**a**) 140 °C—15 min; (**b**) 140 °C—2 h; (**c**) 180 °C—15 min; (**d**) 180 °C—2 h; (**e**) 200 °C—30 min and (**f**) 200 °C—2 h.

Benzyl alcohol is a commonly used solvent in the solvothermal method [22]. Therefore, we used benzyl alcohol as the solvent to synthesize Cs_x_WO_3_ powders under different experimental conditions. Figure 1 shows the XRD patterns of the samples obtained under different reaction conditions using benzyl alcohol as the solvent. As seen in Figure 1a, at a reaction temperature of 120 °C, no Cs_x_WO_3_ phase was formed regardless of the reaction time (15 min, 30 min, 1 h, or 2 h). Comparison with XRD standard cards reveals that the products contain H_x_WO_3_·2H_2_O (JCPDS 40-0693) and WO_3_·0.5H_2_O (JCPDS 84-1851) phases, and with the extension of reaction time, the amount of WO_3_·0.5H_2_O gradually decreases. When the reaction temperature is increased to 140 °C, the products obtained after 15 min still consist of H_x_WO_3_·2H_2_O and WO_3_·0.5H_2_O phases, but with longer reaction times, these phases gradually transform into the Cs_x_WO_3_ phase. After 2 h, a pure Cs_x_WO_3_ phase (JCPDS 81-1224) is obtained (Figure 1b). At a reaction temperature of 180 °C, the products obtained at different reaction times all exhibit a similar crystal structure, with no other impurity phases observed (Figure 1c). The XRD results indicate that at lower temperatures, extending the reaction time leads to the formation of Cs_x_WO_3_, whereas at higher temperatures, Cs_x_WO_3_ can be obtained in a shorter time. When the reaction temperature continues to rise to 200 °C, the sample can still maintain the crystal structure of Cs_x_WO_3_. However, when the reaction time exceeds 1 h, there is a noticeable weakening trend in the peak intensity. From the SEM images in Figure 2, it can be observed that at 200 °C, as the reaction time exceeds 1 h, the particle size gradually decreases. Therefore, we hypothesize that the XRD peak intensities decrease significantly due to a decline in crystallinity. This may arise from structural disorder or lattice strain induced by prolonged synthesis conditions. With the reduction in particle size, the particle surface may undergo amorphization or an increase in internal defects, which leads to a decrease in overall crystallinity, causing a weakening of the XRD peak intensity.

**Figure 3:**
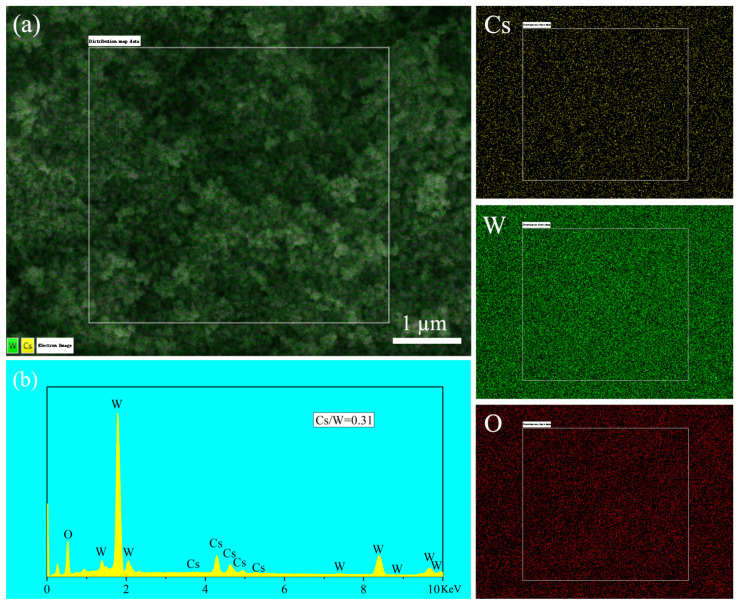
(**a**) Element mapping in the selected area and (**b**) element spectrum of sample synthesized at 140 °C—2 h using benzyl alcohol as solvent.

Figure 2 shows the SEM images of six samples: 140 °C—15 min, 140 °C—2 h, 180 °C—15 min, 180 °C—2 h, 200 °C—30 min, and 200 °C—2 h. From the images, it can be observed that these samples are primarily composed of nanorods with sizes in the tens of nanometers. The particles are well dispersed, and there is no significant difference in particle size and morphology, which is consistent with the XRD results shown in Figure 1. After reacting for 30 min at 200 °C, the particles change from the previous nanorod morphology to irregularly shaped particles with a size of approximately 50 nm. When the reaction time is extended to 2 h, the particle size decreases significantly to around a few nanometers, which is likely to cause a reduction in the XRD peak intensity. Figure 3 shows the EDS results of the sample obtained under the reaction conditions of 140 °C for 2 h. The element mapping in Figure 3a demonstrates a uniform distribution of Cs, W, and O elements within the selected area. The element spectrum in Figure 3b further confirms that no elements other than Cs, W, and O are present in the sample. The Cs/W atomic ratio obtained from the EDS analysis is 0.31, which is close to the intended stoichiometric ratio of 0.33/1.

**Figure 4:**
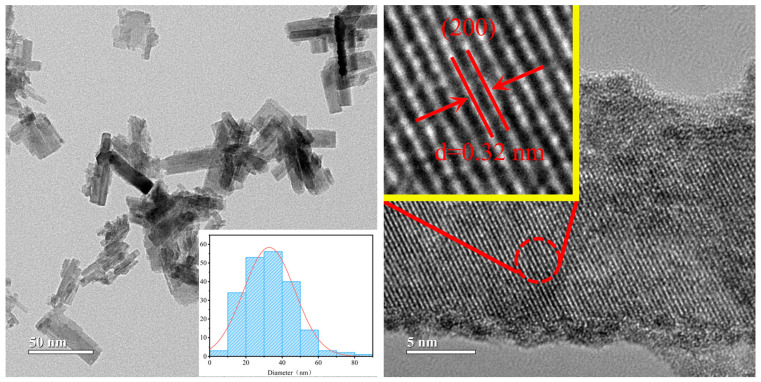
TEM and HRTEM images of the sample synthesized at 140 °C—2 h using benzyl alcohol as solvent.

To further investigate the microstructure of the obtained nanocrystals, we performed an analysis of the samples using transmission electron microscopy (TEM). Figure 4 presents the TEM images of the sample prepared with benzyl alcohol as the solvent at 140 °C for 2 h. The diagram of the nanoparticle size distribution in the TEM image reveals that the sample mainly consists of nanorods with sizes around 40 nm, which is consistent with the results obtained from SEM. Additionally, the interplanar spacing of 0.32 nm observed in the high-resolution lattice image corresponds to the (200) crystal plane spacing of the hexagonal phase of Cs_0.3_WO_3_.

**Figure 5:**
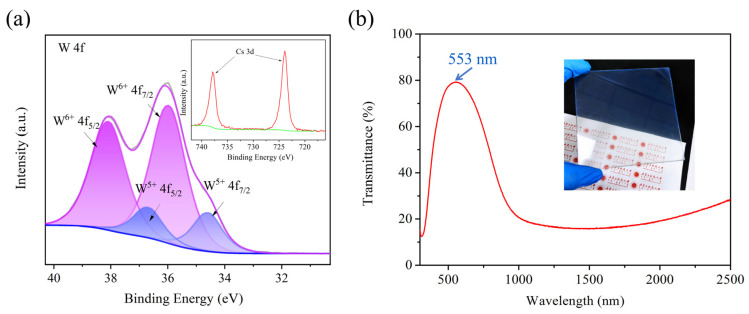
(**a**) W4f core-level XPS spectra of the sample synthesized at 140 °C—2 h using benzyl alcohol as solvent and (**b**) the transmittance curve of its coated glass.

The chemical valence states of Cs_x_WO_3_ nanoparticles were determined using XPS. Figure 5a shows the XPS spectra of the tungsten core level (W4f) and transmittance curve for the sample obtained under the reaction conditions of 140 °C for 2 h in a benzyl alcohol solvent. The fitted spin–orbit doublet peaks of W 4f_7/2_ and W 4f_5/2_ are separated by 2.0 eV, indicating that the W element exists in two different oxidation states. The peaks at 38.1 eV and 36.0 eV correspond to the W^6+^ state, while the peaks at 36.7 eV and 34.6 eV correspond to the W^5+^ state. The typical properties of non-stoichiometric tungsten bronze can be represented as M_x_W^6+^_1−x_W^5+^_x_WO_3_, and our XPS results align with this simplified characterization. The Cs ions provide a large number of free electrons to WO_3_, partially reducing W^6+^ to W^5+^, thereby generating a LSPR effect. From the transmittance curve of the Cs_x_WO_3_-coated glass in Figure 5b, it is evident that the sample exhibits high transmittance in the visible region and low transmittance in the NIR region, indicating the excellent transparent heat-shielding properties of cesium tungsten bronze.

### 3.2. Using Anhydrous Ethanol as Solvent

**Figure 6:**
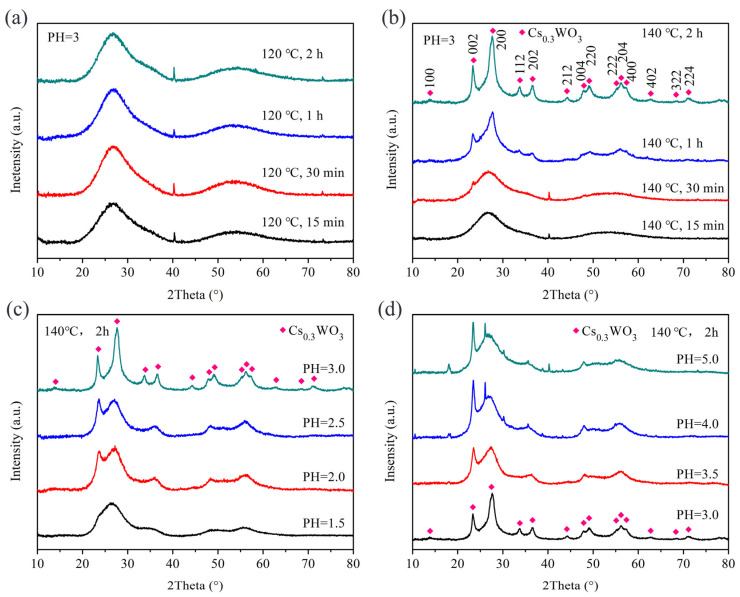
XRD patterns of the samples using anhydrous ethanol as the solvent: (**a**) different treatment time at 120 °C and PH = 3; (**b**) different treatment time at 140 °C and PH = 3; (**c**) different PH (≤3) at 140 °C, 2 h and (**d**) different PH (≥3) at 140 °C, 2 h.

Ethanol is an environmentally friendly solvent with low toxicity. Compared with benzyl alcohol, it is more cost-effective, safer to handle in experiments, and more environmentally friendly [23]. Therefore, we also used anhydrous ethanol as the solvent to prepare Cs_x_WO_3_. During the preparation process, we found that the pH of the ethanol solvent significantly affected the reaction, and Cs_x_WO_3_ samples could only be obtained under an appropriate pH value. Figure 6 presents the XRD patterns of the products obtained under different reaction conditions using anhydrous ethanol as the solvent. As shown in Figure 6a, when the pH is 3, no Cs_x_WO_3_ crystals are formed at a reaction temperature of 120 °C, regardless of the reaction time. However, when the reaction temperature is increased to 140 °C, Cs_x_WO_3_ begins to form gradually with the extension of the reaction time (JCPDS 81-1224). After 2 h of reaction, well-formed Cs_x_WO_3_ crystals are obtained (Figure 6b). Subsequently, we conducted reactions at a reaction temperature of 140 °C for 2 h under different pH conditions. As shown in Figure 6c,d, well-formed Cs_x_WO_3_ crystals are only obtained when the pH is 3. When the pH is either lower than 3 or higher than 3, no well-formed Cs_x_WO_3_ is observed. This may be because, after WCl_6_ hydrolyzes in the solvent, an inappropriate pH causes excess Cl^−^ and H^+^ ions to induce the formation of non-target products, or OH^−^ may react with W^6+^ to form tungsten salts.

**Figure 7:**
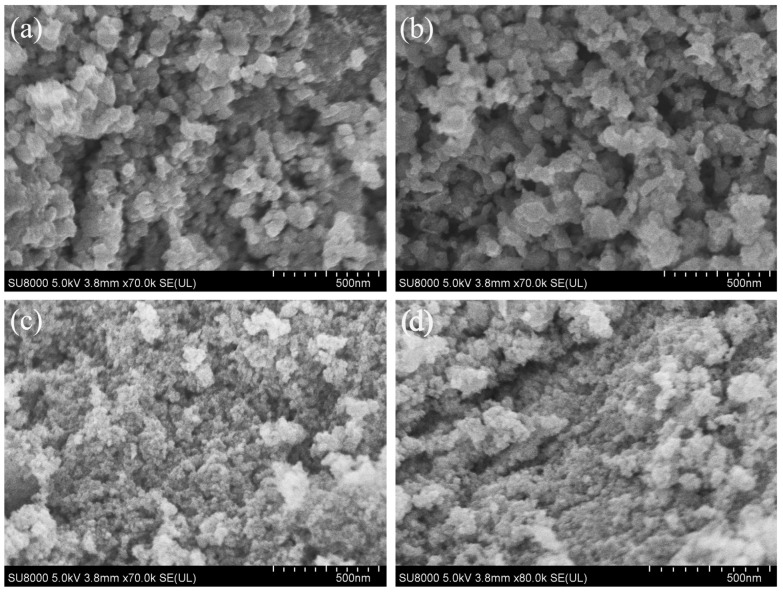
SEM images of four samples using anhydrous ethanol as the solvent: (**a**) PH = 3, 120 °C—2 h, (**b**) PH = 3, 140 °C—2 h, (**c**) PH = 2, 140 °C—2 h, and (**d**) PH = 4, 140 °C—2 h.

**Figure 8:**
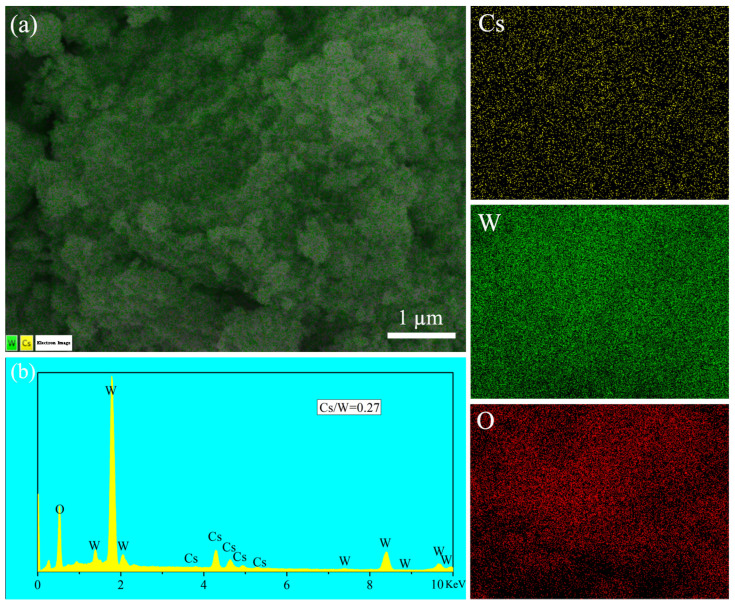
(**a**) Element mapping in the selected area and (**b**) element spectrum of sample synthesized at PH = 3, 140 °C—2 h using anhydrous ethanol as the solvent.

Figure 7 shows the SEM images of four samples obtained under different reaction conditions using anhydrous ethanol as the solvent. Unlike the nanorod-like particles obtained in Figure 3 with benzyl alcohol as the solvent, the products obtained using ethanol as the solvent consist of irregularly shaped particles. From images Figure 7a,b, it can be seen that when pH = 3, the particle size is around tens of nanometers; whereas when pH = 2 or pH = 4, the particle size of the resulting samples is significantly smaller, approximately a few nanometers (Figure 7c,d). This is likely because, at pH = 3, the solubility of the precursor, surface charge state, and reaction kinetics reach an optimal balance, promoting moderate nucleation and allowing particle growth, leading to the formation of Cs_x_WO_3_ particles in the tens of nanometer range. When the pH deviates from 3, rapid nucleation or restricted growth results in a significant reduction in particle size. Figure 8 shows the element mapping and element spectrum of the sample synthesized at pH = 3, 140 °C for 2 h. As seen in Figure 8a, the elements Cs, W, and O are uniformly distributed within the selected region. In the element spectrum shown in Figure 6b, no elements other than Cs, W, and O are detected. The EDS elemental analysis reveals a Cs/W atomic ratio of 0.27:1, with the Cs content being lower than that of the sample obtained using benzyl alcohol as the solvent. This may be due to the lower dielectric constant of benzyl alcohol, which makes it easier for Cs^+^ to approach the WO_3_ surface and enter the lattice, resulting in a higher x value.

**Figure 9:**
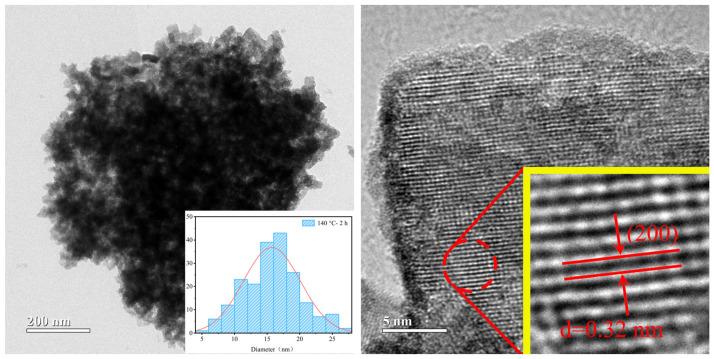
TEM and HRTEM images of the sample synthesized at 140 °C—2 h using anhydrous ethanol as solvent.

The TEM characterization results in Figure 9 demonstrate that when synthesized using anhydrous ethanol as the solvent under 140 °C for 2 h, the obtained sample formed nanoparticles with a relatively uniform size distribution, with particle sizes around a few tens of nanometers. The high-resolution TEM (HRTEM) image reveals clearly visible lattice fringes with an interplanar spacing of 0.32 nm, corresponding to the (200) crystal plane spacing of hexagonal Cs_x_WO_3_.

**Figure 10:**
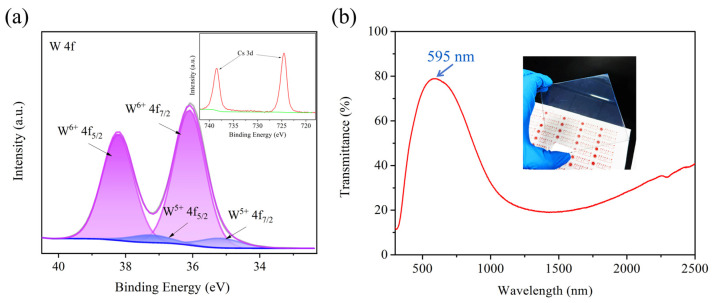
(**a**) W4f core-level XPS spectra of the sample synthesized at 140 °C—2 h using anhydrous ethanol as solvent and (**b**) the transmittance curve of its coated glass.

Figure 10 shows the XPS results and transmittance curve of the sample synthesized at 140 °C for 2 h using ethanol as the solvent. The W4f_7/2_ and W4f_5/2_ spin–orbit doublets are separated by 2.1 eV, with peaks at 38.2 eV and 36.1 eV representing the W^6+^ valence state, and peaks at 37.2 eV and 35.2 eV corresponding to the W^5+^ valence state. The transmittance curve of the coated glass in Figure 10b exhibits a similar trend to that in Figure 5b, but with a noticeable redshift in the visible region, where the peak is at 595 nm compared with 553 nm in Figure 5b. Additionally, the NIR transmittance in the range of 1500–2500 nm is higher than that of the sample synthesized with benzyl alcohol as the solvent. A possible cause for this phenomenon is the Cs content in the sample. Yao et al.’s research showed that samples with lower Cs content exhibited a more pronounced redshift in their transmittance curves [17], which is consistent with our experimental results.

## 4. Conclusions

In this study, we developed a microwave-assisted solvothermal synthesis strategy for the rapid and controllable preparation of Cs_x_WO_3_ nanoparticles under mild conditions. By optimizing reaction parameters, Cs_x_WO_3_ nanoparticles were successfully synthesized at significantly reduced temperatures and ambient pressure. Using benzyl alcohol as the solvent, well-crystallized Cs_x_WO_3_ nanorods were obtained within 2 h at 140 °C or 15 min at 180 °C, while anhydrous ethanol (in actual production, the commonly used 95% ethanol is sufficient) enabled synthesis at 140 °C within 2 h. This approach eliminates the need for high-pressure reactors, mitigates safety risks, and enhances energy efficiency compared with traditional methods. The resulting nanoparticles retained excellent NIR shielding performance and high visible-light transparency, demonstrating their suitability for energy-saving applications such as smart windows. Notably, the solvent choice influenced particle morphology: benzyl alcohol yielded uniform nanorods, whereas ethanol produced irregular nanoparticles. pH optimization in ethanol-based synthesis (pH = 3) was critical for achieving phase-pure Cs_x_WO_3_, highlighting the importance of reaction kinetics and precursor solubility control. The scalable and environmentally friendly nature of this synthesis method positions Cs_x_WO_3_ as a promising candidate for advancing energy-efficient technologies. This work provides a practical pathway for the safe and rapid production of functional nanomaterials, accelerating their industrial adoption in climate control, solar management, and carbon mitigation applications.

## Data Availability

The data that support the findings of this study are available from the corresponding author upon reasonable request.
